# Corpuls CPR Generates Higher Mean Arterial Pressure Than LUCAS II in a Pig Model of Cardiac Arrest

**DOI:** 10.1155/2017/5470406

**Published:** 2017-12-17

**Authors:** S. Eichhorn, A. Mendoza, A. Prinzing, A. Stroh, L. Xinghai, M. Polski, M. Heller, H. Lahm, E. Wolf, R. Lange, M. Krane

**Affiliations:** ^1^Institute for Translational Cardiac Surgery (INSURE), Department of Cardiovascular Surgery, German Heart Center Munich, Technische Universität München, Munich, Germany; ^2^Fakultät für Informatik, Robotics and Embedded Systems, Technische Universität München, Munich, Germany; ^3^GS Elektromedizinische Geräte GmbH, Kaufering, Germany; ^4^Gene Center, LMU Munich, Munich, Germany; ^5^German Center for Cardiovascular Research (DZHK), Partner Site Munich Heart Alliance, Munich, Germany

## Abstract

According to the European Resuscitation Council guidelines, the use of mechanical chest compression devices is a reasonable alternative in situations where manual chest compression is impractical or compromises provider safety. The aim of this study is to compare the performance of a recently developed chest compression device (Corpuls CPR) with an established system (LUCAS II) in a pig model.* Methods*. Pigs (*n* = 5/group) in provoked ventricular fibrillation were left untreated for 5 minutes, after which 15 min of cardiopulmonary resuscitation was performed with chest compressions. After 15 min, defibrillation was performed every 2 min if necessary, and up to 3 doses of adrenaline were given. If there was no return of spontaneous circulation after 25 min, the experiment was terminated. Coronary perfusion pressure, carotid blood flow, end-expiratory CO_2_, regional oxygen saturation by near infrared spectroscopy, blood gas, and local organ perfusion with fluorescent labelled microspheres were measured at baseline and during resuscitation.* Results*. Animals treated with Corpuls CPR had significantly higher mean arterial pressures during resuscitation, along with a detectable trend of greater carotid blood flow and organ perfusion.* Conclusion*. Chest compressions with the Corpuls CPR device generated significantly higher mean arterial pressures than compressions performed with the LUCAS II device.

## 1. Introduction

Chest compressions are crucial for maintaining coronary and cerebral perfusion during cardiac arrest. The efficiency of manual chest compressions during cardiopulmonary resuscitation (CPR) decreases over time [1, 2], and it is difficult to perform efficient chest compressions during transportation or during interventional procedures, for example, in a catheter lab.

In order to address these problems, a variety of devices that perform mechanical chest compressions have been developed and tested in animal experiments, experimental investigations using manikins, and clinical studies [3–7].

The 2015 European Resuscitation Council (ERC) guidelines for CPR recommend mechanical chest compression devices as a reasonable alternative in situations where delivery of high performance chest compressions is impeded or would compromise provider safety [8].

These devices should offer maximal flexibility for adaptation to the individual constitution of the patient, as well as adequate battery capacity, low weight, and mechanical stability that allows compressions of sufficient depth even at high chest stiffness values. The LUCAS II device is currently one of the most widely used chest compression machines in clinical practice. This device has a closed frame that surrounds the patient to provide a maximum of stability.

Corpuls CPR (GS Elektromedizinische Geräte G. Stemple GmbH, Kaufering, Germany) is a newly introduced electric device for chest compressions. Compression is generated by a single, flexible, adaptable arm that is locked in a spine board or to a small baseplate positioned under the patient. The device works with a duty cycle of 50% and typically has an average battery capacity of 90 min. It offers an adjustable compression frequency from 80 to 120 compressions/minute and a compression depth of 20–60 mm. The therapy mode can be changed between 30 : 2/15 : 2 and continuous mode. The position of the stamp is checked after each ventilation break or 100 compressions (continuous mode) and compensated if a sunken thorax is detected [9–11].

The aim of the present study is to compare the effects of the performance of this device with the clinically established LUCAS II device in a pig model of cardiac arrest.

## 2. Materials and Methods

A total of 10 female German Landrace pigs weighing 25 ± 2.5 kg were used in the study. All animals received care in compliance with the European convention for the protection of vertebrate animals used for experimental and other scientific purposes. The study protocol was approved by the local government (Regierung von Oberbayern, Ref. number 55.2-1-54-2532-205-2013).

Ketamine (15 mg/kg), azaperone (2 mg/kg), and atropine (0.02 mg/kg) were injected intramuscularly (neck region according to Swindle and Smith [12]) for premedication. The pigs were placed in a supine position, and endotracheal intubation via tracheotomy was performed after intravenous bolus injection of propofol (10 mg/kg) and fentanyl (0.04 mg). Anesthesia was maintained by continuous infusion of propofol (8 mg/kg/h) and fentanyl (25 *μ*g/kg/h), and intravenous Ringer's solution (10–15 ml/kg/h) was administered to maintain a mean arterial pressure of 80–90 mmHg.

Volume-controlled ventilation (tidal volume 8 to 10 ml/kg, PEEP 5 cm H_2_O, FIO_2_ 0.21 to 0.3, and *P*_max_ 45 mbar) was performed using an Evita II respirator (Draeger, Lübeck, Germany). Oxygen was added to maintain saturation >95%. End-expiratory CO_2_ (mainstream technique) and oxygen saturation (sensor placed at the tongue) as well as the electrocardiogram (ECG) based on pads (Ambu Blue Sensor, Ambu Germany, Friedheim) were monitored by a Corpuls 3 device (GS Elektromedizinische Geräte G. Stemple GmbH, Kaufering, Germany). Respiratory frequency was adjusted to maintain an end-expiratory CO_2_ partial pressure between 35 and 40 mmHg before cardiac arrest. Arterial blood gas samples were taken from the introducer placed in the femoral artery every 15 min during preparation, and the ventilation was adjusted accordingly.

An ultrasonic flow probe (Transonic 202, Ithaca, USA) was placed around the right carotid artery, and a temporary pacemaker wire was inserted into the right ventricle via the right external jugular vein. Additionally, a catheter for sampling aortic blood was placed via the subclavian artery. Micromanometers (Millar-TIP SPC 350, Houston, TX, USA) were placed in the ascending aorta and the right atrium via bilateral femoral cutdown, and a pigtail catheter was placed in the descending aorta from the left femoral arteria. Blood pressure was monitored at the femoral artery with a fluid-filled line and pressure transducer (Xtrans, PVB Codan Critical Care, Forstinning, Germany).

Near infrared spectroscopy (NIRS) sensors (Equanox, Nonin, North Plymouth, MN, USA) were placed in the frontal region of the scull, in submental position, and on the lower left quadrant of the abdomen for monitoring regional oxygen saturation (% RO_2_) in accordance with the protocol for humans weighing <40 kg (NO80004CB). Each area was shaved and cleaned thoroughly with isopropanol prior to placement of the sensors.

Hemodynamic data were recorded continuously at a frequency of 1 kHz with Powerlab 8.0 (AD Instruments, Oxford, UK).

Fluorescent labelled microspheres (Molecular Probes, 15 *μ*m, Life Technologies, Eugene, OR, USA) were used to measure local organ perfusion at baseline and after 5 min of resuscitation as follows: 10^6^ microspheres/10 kg body weight were injected via the pigtail catheter, which was directly above the aortic valve, and reference samples were taken via syringe pump over a catheter placed in the descending aorta at a rate of 10 mL/min for 4 minutes.

**Figure 1:**
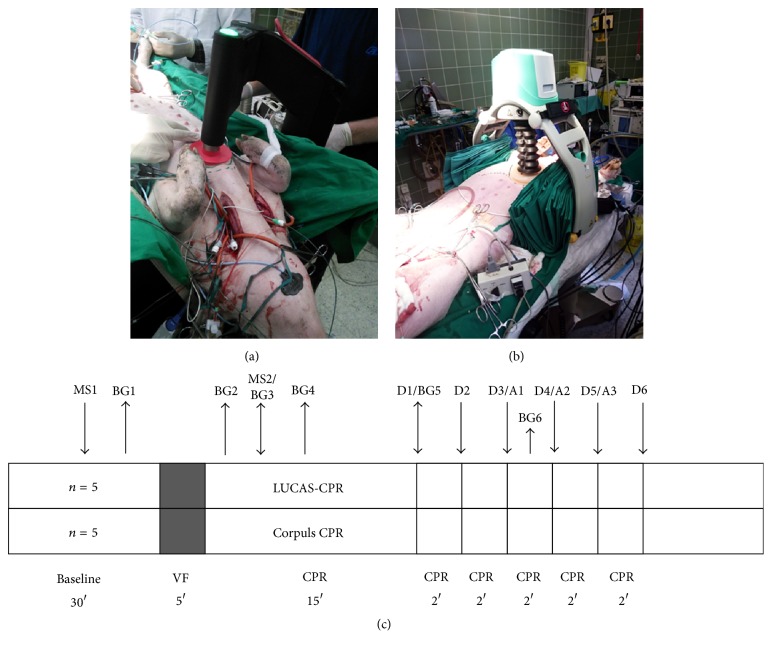
(a) Pig in a v-shaped board during treatment with the Corpuls CPR. (b) Pig fixed with cushions in the LUCAS II. (c) Description of the study protocol, MS: microsphere injection, BG: blood gas sample, D: defibrillation, A: administration of adrenaline.

Before initiation of ventricular fibrillation, the pigs were randomised into two groups, Corpuls CPR (CCPR) or LUCAS II CPR, by a sealed envelope method. Pigs that received CCPR were secured to a v-shaped board prior to induction of ventricular fibrillation and those that were treated with the LUCAS II device were secured inside the device by padding on the left and right sides between the pig and the load frame (Figure 1).

Resuscitation was performed according to the protocol outlined in Figure 1. Ventricular fibrillation was induced by a 14 V direct current pulse via the pacemaker and the pigs were left untreated for 5 min. The respirator was disconnected and the infusion of propofol and fentanyl was stopped. After 5 min, CPR was initiated. Both devices were operating with 100 compressions/min in continuous mode and a compression depth of 50 mm with a duty cycle 50%. 10 ventilations per minute were performed with a Ruben bag supplied with 100% oxygen.

The fluorescent microspheres were injected at 5 min of resuscitation. After 15 min of continuous resuscitation the compressions were stopped and defibrillation was performed with a 150 J biphasic impulse in cases of ongoing ventricular fibrillation. Compressions were then resumed for 2 min, and up to 3 doses of epinephrine (0.01 mg/kg mg) were given after 3 cycles of 2 min chest compression after defibrillation. If there was no return of spontaneous circulation (ROSC) after 6 times of defibrillation and 3 doses of epinephrine the experiment was stopped. Necropsies were performed with special attention to compression-related chest injuries that might have affected ROSC (pericardial effusion, pneumothorax, hemothorax).

Blood gas samples were collected every 5 min after initiation of cardiac arrest; the sampling included lactate measurement by Siemens Rapid Point 500 (Siemens, Erlangen, Germany). Cardiac perfusion pressure was calculated according to the end diastolic method [13] using an average of 10 compression cycles. Mean arterial pressure (MAP), mean carotid blood flow (CBF), regional oxygen saturation (% RO_2_) by NIRS, and end-expiratory CO_2_ (ETCO_2_) were also measured to evaluate the performance of the resuscitation devices. The data was recorded continuously using Powerlab and Labchart (AD Instruments, Sydney, Australia). For evaluation, baseline data before initiation of ventricular fibrillation, after 5 minutes of cardiac arrest, and during resuscitation (1; 5; 10; 15; 20 minutes) were taken.

Normal distribution of the data was analyzed using the Kolmogorov–Smirnov test. Comparison of variables between the 2 groups was performed with Student's *t*-test for unpaired observations. A *p* value of <0.05 was regarded as an indicator of statistically significant differences between the groups. All statistical analyses were carried out using IBM SPSS V20.

## 3. Results

MAP measured at the femoral artery was significantly higher during CCPR throughout resuscitation period (MAP = approximately 43 mmHg, CCPR, versus 23 mmHG, LUCAS II).

CBF declined to 30% of the baseline value at the beginning of resuscitation and decreased to 20% of the initial value at the end of the resuscitation period. CBF was significantly higher in the CCPR group after 20 min of resuscitation and after administration of vasopressors. Detailed results are shown in Table 2 and Figure 3.

There were no significant differences between the groups in baseline ETCO_2_, MAP, CPP, CBF, % RO_2_, lactate levels, or degree of local organ perfusion by microspheres. Detailed results are presented in Table 1.

CPP during resuscitation was similar between the groups, measuring approximately 20 mmHg for both.

There was also no significant difference between groups in the degree of local organ perfusion by microspheres after 5 min of resuscitation. Cerebral perfusion levels of 23 ± 7.5% of baseline were recorded for the CCPR group, compared to 12.6 ± 6.7% of baseline in the LUCAS II group. Cardiac perfusion of 26 ± 9% of baseline was detected in the CCPR group, compared to a cardiac perfusion of 17 ± 2% of baseline in the LUCAS II group. Renal perfusion decreased to 19.4 ± 7.8% of baseline in the CCPR group and to 20.2 ± 4.9% of baseline in the LUCAS II group. Hepatic perfusion also declined to 12.1 ± 7.3% of baseline in the CCPR group and 8.9 ± 2.9% in the LUCAS II group.

**Figure 2:**
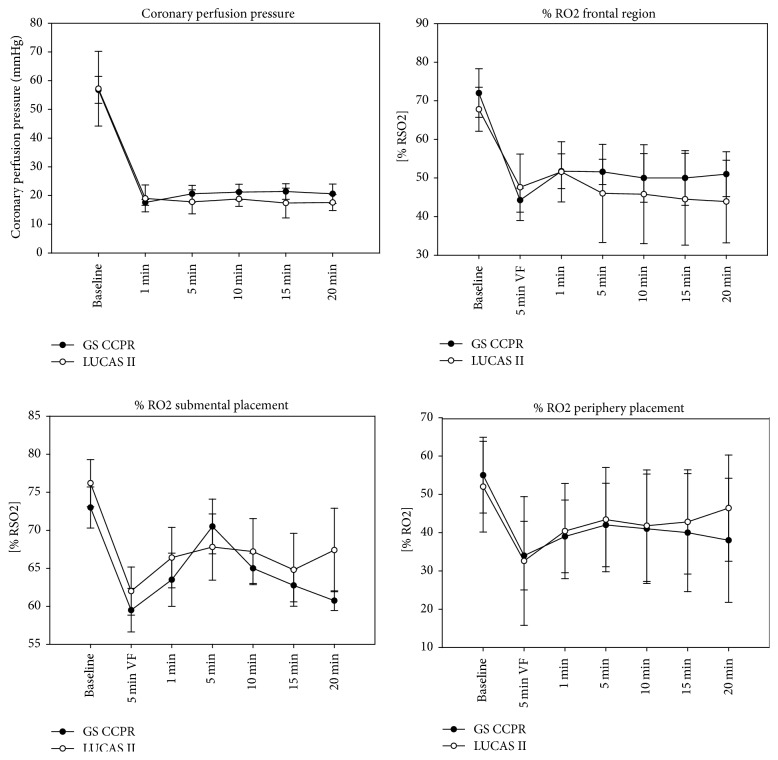
Coronary perfusion pressure and regional oxygen saturation (frontal region, submental placement, periphery placement) during resuscitation. No significant difference was detectable over the whole period (*p* > 0.05). Submental placement seems to produce the highest changes in % RO_2_ between the different measurement points in our pig model.

**Figure 3:**
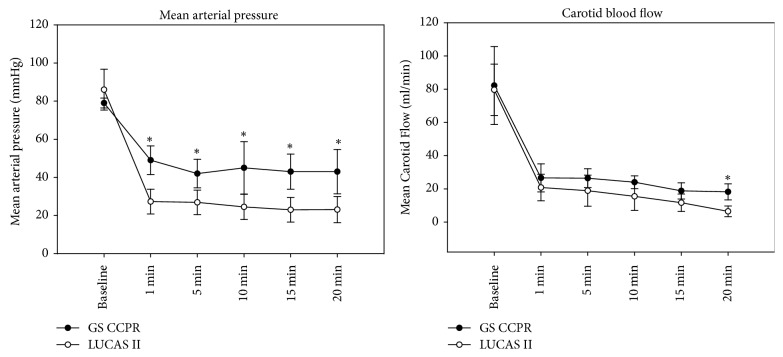
Mean arterial pressure and carotid blood flow during resuscitation (^**∗**^*p* < 0.05). Corpuls CPR is generating a significantly higher mean arterial pressure during the whole resuscitation period. Carotid blood flow seems to be higher by trend during the resuscitation period; after 20 minutes of resuscitation a significant difference could be detected.

NIRS measurements did not differ significantly between the groups. Measurements taken with the submental sensor indicated a decrease in oxygen saturation of about 20% from baseline after 5 min of cardiac arrest. Regional oxygen saturation increased by approximately 10% during CPR. Measurements obtained by probes in the frontal position showed a decrease in oxygen saturation to approximately 65% of baseline at 5 min after cardiac arrest and this value increased to approximately 70% of baseline during CPR. The peripheral sensors indicated regional oxygen saturation of approximately 80% of baseline at 5 min after arrest; this increased to 90% of baseline during CPR. There were no significant differences in % RO_2_ between groups at any of the sensor positions. Detailed results are shown in Figure 2.

Finally, there were no significant differences in ETCO_2_, potassium levels, lactate levels, or pH values between the two groups. There was a slight increase of CO_2_ detected after 5 min of resuscitation, which decreased again (Table 2) during resuscitation. pH values were decreased in both groups from a baseline of approximately 7.4 to 7.25 after 20 min of resuscitation. Potassium levels increased during treatment, from approximately 4.2 mmol/L at baseline to 6.8 mmol/L at the end of the experiment. The lactate levels increased from approximately 1.55 mmol/L at baseline to 8.2 mmol/L at the end of the experiment (Table 3). Three animals from each group received defibrillation; 2 animals per group were asystolic after 15 min of CPR; and no pig in either group had ROSC. At autopsy, in the macroscopic inspection of the opened chest we detected no rib fractures that caused harmful injuries influencing ROSC like pneumothorax, hemothorax, or pericardial effusions in either group.

## 4. Discussion

The haemodynamic parameters at baseline and during resuscitation in our experiments corresponded to the results of previous evaluations of mechanical resuscitation devices. Halperin et al. [6] generated CPP between 14 and 21 mmHg, cerebral flow of approximately 0.2 mL/min/g, and MAP of approximately 36 mmHg during CPR using a load distributing band (Autopulse). Steen et al. [7], in an evaluation of a LUCAS device, measured CBF of approximately 30% of baseline and MAP of approximately 40 mmHg, and Liao et al. [14] reported CPP of >20 mmHg and a CBF of approximately 30 to 35% of baseline during CPR with the LUCAS II device in pig models. The LUCAS II system is presently the most widely used mechanical chest compression system, and a number of experiments* and *clinical trials have been performed to evaluate its efficacy [15–20]. Therefore, we used the LUCAS II system as the reference device for comparison with the Corpuls CPR device.

We found that the Corpuls device was able to generate a significantly higher MAP than the LUCAS device. There was also a trend towards greater CBF and improved local organ perfusion with the Corpuls device, although this was not statistically significant.

The ability of CCPR to generate higher MAP and CBF might be related to a difference in the compression waveform [9] or to the different shape of the chest compression plate. Neither of the compression plates that were used in our experiments has a feature for active chest recoil, and the diameters of the contact areas are comparable. Thus, the difference in flow and pressure is probably not related to the compression plate.

On the other hand, CCPR produces a slightly more trapezoidal compression waveform than the LUCAS II device. Using an artificial chest model with integrated blood flow we could measure the compression waveform of the two devices and produce analogue results concerning MAP and arterial blood flow comparing the LUCAS II and the Corpuls CPR [9]. Kramer-Johansen et al. [1] have reported similar results secondary to modifications of the compression waveform in a computer simulation as well as in a pig model. There are two effects that are mentioned in literature causing blood circulation during CPR, the direct cardiac compression and the thoracic pump theory [21, 22]. If the flow is predominantly created by the thoracic pump, a more trapezoid compression waveform with prolonged compression time will increase the flow. In the chest of a pig the ventricles are embedded with lung tissue from all sides, and the compressions given to the thorax are affecting the heart and the big vessels much more by the thoracic pump mechanism than by the direct compression mechanism in humans [14]. This might also be an explanation that although having similar cardiac perfusion pressures in the group of Corpuls CPR, a higher MAP could be generated.

In a study performed by Paradis et al. [23] only patients with a CPP of 15 mmHg or higher reached ROSC. Similar findings for pigs were obtained by Steen et al. [7]. In our study, CPP values of >15 mmHg were reached in all animals during CPR, and there were no signs of pericardial effusion or pneumothorax at necropsy that would have been affecting ROSC. However, none of the animals had ROSC. In a pig model with a comparable study design Liao et al. reached a ROSC rate of 100% using the LUCAS II device [14]. Unlike our examination, they used a suction cup that provides active chest recoil. Consecutively this resulted in significantly lower pressure in the right atrium during the decompression phase and additionally in a higher intrathoracic aortic pressure during the end decompression phase. We used the german model of LUCAS II with holes in the suction cup, which allows no active chest recoil in the decompression phase.

Another difference from the examination of Liao et al. is the animals they used in their study. In contrast to us, they used Swedish domestic pigs with a mean weight of 31 kg; we were using German Landrace pigs with a mean weight of 25 kg. There could be an influence caused by the breed or more probably due to the lower weight of the animals. In contrast to their study, we also had found pigs having asystole after 15 minutes of CPR in both groups. Also the vasoconstrictive effect of the administered adrenaline could not be detected significantly. These two additional findings can also be related to the compression without active chest recoil, or breed and weight.

Our protocol was designed to characterize the differences in flow and pressure related to the chest compression device, with minimized influence of defibrillation or drugs. That is why we chose to allow a long interval of ventricular fibrillation (5 min), compared to prior investigations in which the untreated interval was only 60 to 90 s [6, 7, 24]. With this consideration, we accepted a lower chance of ROSC in exchange for study conditions that favoured evaluation of the influence of different compression devices on haemodynamic performance.

Additionally, in our protocol, the first 15 min of resuscitation included only mechanical chest compressions and Ruben bag ventilation. No additional treatments, that is, defibrillation or medications, were provided during this period. It has been shown that survival rates and neurological outcome are worse after a longer duration of fibrillation in men and in pig [25–28], and early defibrillation and administration of antiarrhythmic drugs or vasopressors would have most likely increased the rate of ROSC in our study.

Peak forces of up to 600 N have been reported during chest compression [29–31]. The frame of a resuscitation device must be very rigid to reach a compression depth of 50 mm. Examination of the recorded data of the LUCAS II device used in 59 cases of cardiac arrest by Beesems et al. [32] showed that the LUCAS II device was able to generate sufficient compression of 50 mm in all cases.

There was initial concern that the flexible open frame of the Corpuls CPR device would not have sufficient rigidity to ensure proper compression depths in different chest profiles. In previous experiments based on a mechanical chest model [9] and in the animal experiments, there were no difficulties reaching the recommended compression depth of 50 mm with the Corpuls device in any case, and no immoderate bending or moving of the stamp on the compression area was noted.

Several studies have been performed to determine the usefulness of NIRS as a neuromonitoring tool for prediction of outcomes, detection of ROSC, or evaluation of the quality of brain perfusion during CPR [33]. We found a significant difference in % RO_2_ between baseline, after 5 min of cardiac arrest, and after 5 min of CPR. We also found a very high level of interindividual deviation of the absolute values of % RO_2_, which might indicate that the chosen sensor system that was designed for the human brain is not suitable for examination in pigs.

We were using the smallest available paediatric sensors for NIRS monitoring, which are designed for human use. The dimensions of the skull and brain of the pig are anatomically different from those of the human, and this might explain the large interindividual spread of the recorded values. In our opinion, in experiments with a pig model, submental sensor placement is preferable to frontal region placement for monitoring cerebral perfusion. We found that the most impressive changes with the least interindividual deviation were detected when the sensors were placed in the submental position. This might be due to better adaptation of the sensor to the tissue in the submental region, as the plane area for correct sensor placement in the frontal region is limited.

We were able to use NIRS to measure baseline oxygen saturation values before cardiac arrest. The usefulness of information obtained from NIRS measurements during resuscitation might be limited without prior baseline measurements, as is often the case in real world emergency situations. Nonetheless, our results support the conceptual premise that regional oxygen saturation can detect changes in cerebral perfusion brain during CPR. Whether this method is suitable to predict ROSC or the neurological outcome, as other authors have suggested [34, 35], or whether it can provide further information regarding the quality of CPR cannot be finally answered based on the design and results of our study. Further investigations focusing especially on the use of NIRS in CPR are necessary.

## 5. Conclusion

In conclusion, we found that the Corpuls CPR device was equivalent or superior to the LUCAS II system in terms of blood pressure and flow during resuscitation in a pig model of cardiac arrest. Chest compressions with the Corpuls CPR device generated significantly higher MAP compared to compressions with a LUCAS II device.

## Figures and Tables

**Table 1:** Baseline values in the two groups showed no statistical difference.

	MAP [mmHg]	CPP [mmHg]	Av. CBF [ml/min]	ET CO_2_ [mmHg]	PH	Lac [mmol/L]
CCPR	79 ± 2.6	56.8 ± 4.7	82 ± 23	37.9 ± 3.1	7,4 ± 0.02	1.5 ± 0,38
LUCAS II	82 ± 12.1	57.2 ± 13.1	79.6 ± 15.4	37.2 ± 0.75	7.4 ± 0.04	1.59 ± 0.34

Local perfusion	Brain [ml/min 100 g]	Heart [ml/min 100 g]	Kidney [ml/min 100 g]	Liver [ml/min 100 g]		

CCPR	36.6 ± 5.08	93.8 ± 18.68	236 ± 38.2	28.6 ± 8.55		
LUCAS II	36 ± 3.63	110.4 ± 6.53	230.6 ± 48.2	25 ± 3.3		

MAP: mean arterial pressure; CPP: cerebral perfusion pressure; Av. CBF: average cerebral blood flow; ET CO_2_: end-tidal carbon dioxide; Lac: lactate; CCPR: Corpuls CPR.

**Table 2:** Mean arterial pressure, local perfusion, Et CO_2_, and carotid blood flow during resuscitation.

MAP [mmHg]	MAP 1 minute	MAP 5 minutes	MAP 10 minutes	MAP 15 minutes	MAP 20 minutes
CCPR	49.4 ± 7.58	42.2 ± 7.11	45.6 ± 13.7	43.4 ± 9.2	43.2 ± 10.7
LUCAS II	25.9 ± 6.53	25,54 ± 6.53	23.0 ± 6.7	21.6 ± 6.4	21.9 ± 6.6

CBF [ml/min]	CBF 1 minute	CBF 5 minutes	CBF 10 minutes	CBF 15 minutes	CBF 20 minutes

CCPR	26.6 ± 8.45	26.4 ± 5.68	24.0 ± 3.85	18.8 ± 4.7	18.2 ± 4.8
LUCAS II	20.82 ± 8.01	18.9 ± 9.34	15.5 ± 8.43	11.66 ± 5.21	6.48 ± 3.23

Et CO_2_ [mmHg]	Et CO_2_ 1 minute	Et CO_2_ 5 minutes	Et CO_2_ 10 minutes	Et CO_2_ 15 minutes	Et CO_2_ 20 minutes

CCPR	22.62 ± 9.27	34.6 ± 25.76	23.1 ± 12.64	17.68 ± 7.08	15.94 ± 8.42
LUCAS II	22.58 ± 5.49	22.88 ± 9.31	22.84 ± 10.5	19.88 ± 9.96	15.9 ± 4.17

Local perfusion at 5 min	Brain [ml/min 100 g]	Heart [ml/min 100 g]	Kidney [ml/min 100 g]	Liver [ml/min 100 g]	

CCPR	8.24 ± 2.17	25 ± 8.39	45.8 ± 18.5	3.4 ± 2.06	
LUCAS II	4.54 ± 2.41	18.8 ± 1.94	46.6 ± 11.3	2.24 ± 0.73	

MAP: mean arterial pressure. Mean arterial pressure was significantly higher in the Corpuls CPR group throughout the entire resuscitation period. CBF: carotid blood flow. Carotid blood flow was significantly higher in the Corpuls CPR group at 20 min. CCPR: Corpuls CPR.

**Table 3:** Blood gas values at baseline and during resuscitation (mean and SD).

pH value	Baseline	1 min resuscitation	5 min resuscitation	10 min resuscitation	15 min resuscitation	20 min resuscitation
CCPR	7.41 ± 0.02	7.44 ± 0.1	7.37 ± 0.07	7.23 ± 0.12	7.19 ± 0.21	7.25 ± 0.16
LUCAS II	7.42 ± 0.04	6.86 ± 1.18	7.39 ± 0.14	7.35 ± 0.1	7.34 ± 0.11	7.31 ± 0.08

Potassium [mmol/l]	Baseline	1 min resuscitation	5 min resuscitation	10 min resuscitation	15 min resuscitation	20 min resuscitation

CCPR	4.2 ± 0.28	5.02 ± 0.95	6.39 ± 0.34	6.06 ± 0.39	6.15 ± 0.38	6.76 ± 0.85
LUCAS II	4.11 ± 0.24	4.77 ± 0.49	6.72 ± 0.54	6.34 ± 0.83	6.21 ± 0.8	6.81 ± 0.75

Lactate [mmol/l]	Baseline	1 min resuscitation	5 min resuscitation	10 min resuscitation	15 min resuscitation	20 min resuscitation

CCPR	1.52 ± 0.38	2.91 ± 1.97	5.68 ± 2.41	6.87 ± 1.88	7.65 ± 1.48	8.69 ± 1.76
LUCAS II	1.59 ± 0.34	2.1 ± 0.35	5.35 ± 1.44	6.27 ± 1.3	6.6 ± 1.19	8.1 ± 1.35

## References

[1] Kramer-Johansen J., Myklebust H., Wik L. (2006). Quality of out-of-hospital cardiopulmonary resuscitation with real time automated feedback: a prospective interventional study. *Resuscitation*.

[2] Wik L., Kramer-Johansen J., Myklebust H. (2005). Quality of cardiopulmonary resuscitation during out-of-hospital cardiac arrest. *The Journal of the American Medical Association*.

[3] Putzer G., Braun P., Zimmermann A. (2013). LUCAS compared to manual cardiopulmonary resuscitation is more effective during helicopter rescue - A prospective, randomized, cross-over manikin study. *The American Journal of Emergency Medicine*.

[4] Harrison-Paul R. (2007). A history of mechanical devices for providing external chest compressions. *Resuscitation*.

[5] Timerman S., Cardoso L. F., Ramires J. A. F., Halperin H. (2004). Improved hemodynamic performance with a novel chest compression device during treatment of in-hospital cardiac arrest. *Resuscitation*.

[6] Halperin H. R., Paradis N., Ornato J. P. (2004). Cardiopulmonary resuscitation with a novel chest compression device in a porcine model of cardiac arrest: Improved hemodynamics and mechanisms. *Journal of the American College of Cardiology*.

[7] Steen S., Liao Q., Pierre L., Paskevicius A., Sjöberg T. (2002). Evaluation of LUCAS, a new device for automatic mechanical compression and active decompression resuscitation. *Resuscitation*.

[8] Monsieurs K., De Cauwer H., Bossaert L. (1996). O-8 Feeling for the carotid pulse: Is five seconds enough?. *Resuscitation*.

[9] Eichhorn S., Mendoza Garcia A., Polski M. (2017). Corpuls cpr resuscitation device generates superior emulated flows and pressures than LUCAS II in a mechanical thorax model. *Australasian Physical & Engineering Sciences in Medicine*.

[10] Gmbh GEGCorpuls CPR. http://www.corpuls.com.

[11] Prinzing A., Eichhorn S., Deutsch M. A. (2015). Cardiopulmonary resuscitation using electrically driven devices: a review. *Journal of Thoracic Disease*.

[12] Swindle M. M., Smith A. C. (2013). Best practices for performing experimental surgery in swine. *Journal of Investigative Surgery*.

[13] Otlewski M. P., Geddes L. A., Pargett M., Babbs C. F. (2009). Methods for calculating coronary perfusion pressure during CPR. *Cardiovascular Engineering*.

[14] Liao Q., Sjoberg T., Paskevicius A., Wohlfart B., Steen S. (2010). Manual versus mechanical cardiopulmonary resuscitation. An experimental study in pigs. *BMC Cardiovascular Disorders*.

[15] Gates S., Quinn T., Deakin C. D., Blair L., Couper K., Perkins G. D. (2015). Mechanical chest compression for out of hospital cardiac arrest: Systematic review and meta-analysis. *Resuscitation*.

[16] Gates S., Smith J. L., Ong G. J., Brace S. J., Perkins G. D. (2012). Effectiveness of the LUCAS device for mechanical chest compression after cardiac arrest: Systematic review of experimental, observational and animal studies. *Heart*.

[17] Perkins G. D., Lall R., Quinn T. (2015). Mechanical versus manual chest compression for out-of-hospital cardiac arrest (PARAMEDIC): a pragmatic, cluster randomised controlled trial. *The Lancet*.

[18] Anantharaman V., Ng B. L., Ang S. H. (2017). Prompt use of mechanical cardiopulmonary resuscitation in out-of-hospital cardiac arrest: the MECCA study report. *Singapore Medical Journal*.

[19] Rubertsson S., Lindgren E., Smekal D. (2014). Mechanical chest compressions and simultaneous defibrillation vs conventional cardiopulmonary resuscitation in out-of-hospital cardiac arrest: The LINC randomized trial. *Journal of the American Medical Association*.

[20] Tranberg T., Lassen J. F., Kaltoft A. K. (2015). Quality of cardiopulmonary resuscitation in out-of-hospital cardiac arrest before and after introduction of a mechanical chest compression device, LUCAS-2; a prospective, observational study. *Scandinavian Journal of Trauma, Resuscitation and Emergency Medicine *.

[21] Criley J. M., Niemann J. T., Rosborough J. P., Ung S., Suzuki J. (1981). The heart is a conduit in CPR.. *Critical Care Medicine*.

[22] Kouwenhoven W. B., Jude J. R., Knickerbocker G. G. (1984). Closed-Chest Cardiac Massage. *Journal of the American Medical Association*.

[23] Paradis N. A., Martin G. B., Rivers E. P. (1990). Coronary perfusion pressure and the return of spontaneous circulation in human cardiopulmonary resuscitation. *The Journal of the American Medical Association*.

[24] Wagner H., Madsen Hardig B., Steen S., Sjoberg T., Harnek J., Olivecrona G. K. (2011). Evaluation of coronary blood flow velocity during cardiac arrest with circulation maintained through mechanical chest compressions in a porcine model. *BMC Cardiovascular Disorders*.

[25] Martin T. G., Hawkins N. S., Weigel J. A., Rider D. E., Buckingham B. D. (1988). Initial treatment of ventricular fibrillation: Defibrillation or drug therapy. *The American Journal of Emergency Medicine*.

[26] Weaver W. D., Copass M. K., Bufi D., Ray R., Hallstrom A. P., Cobb L. A. (1984). Improved neurologic recovery and survival after early defibrillation. *Circulation*.

[27] Gu W., Hou X., Li C. (2014). Effect of different resuscitation strategies on post-resuscitation brain damage in a porcine model of prolonged cardiac arrest. *Chinese Medical Journal*.

[28] Indik J. H., Shanmugasundaram M., Allen D. (2009). Predictors of resuscitation outcome in a swine model of VF cardiac arrest: A comparison of VF duration, presence of acute myocardial infarction and VF waveform. *Resuscitation*.

[29] Bankman I. N., Gruben K. G., Halperin H. R., Popel A. S., Guerci A. D., Tsitlik J. E. (1990). Identification of dynamic mechanical parameters of the human chest during manual cardiopulmonary resuscitation. *IEEE Transactions on Biomedical Engineering*.

[30] Gruben K. G., Guerci A. D., Halperin H. R., Popel A. S., Tsitlik J. E. (1993). Sternal force-displacement relationship during cardiopulmonary resuscitation. *Journal of Biomechanical Engineering*.

[31] Neurauter A., Nysæther J., Kramer-Johansen J. (2009). Comparison of mechanical characteristics of the human and porcine chest during cardiopulmonary resuscitation. *Resuscitation*.

[32] Beesems S. G., Hardig B. M., Nilsson A., Koster R. W. (2015). Force and depth of mechanical chest compressions and their relation to chest height and gender in an out-of-hospital setting. *Resuscitation*.

[33] Genbrugge C., Dens J., Meex I. (2016). Regional Cerebral Oximetry during Cardiopulmonary Resuscitation: Useful or Useless?. *The Journal of Emergency Medicine*.

[34] Asim K., Gokhan E., Ozlem B. (2014). Near infrared spectrophotometry (cerebral oximetry) in predicting the return of spontaneous circulation in out-of-hospital cardiac arrest. *The American Journal of Emergency Medicine*.

[35] Parnia S., Nasir A., Shah C., Patel R., Mani A., Richman P. (2012). A feasibility study evaluating the role of cerebral oximetry in predicting return of spontaneous circulation in cardiac arrest. *Resuscitation*.

